# Prevalence of primary HIV-1 drug resistance among antiretroviral-naïve individuals in Togo in 2023: a national study

**DOI:** 10.3389/fpubh.2025.1605763

**Published:** 2025-11-05

**Authors:** Alassane Ouro-Medeli, Roméo M. Togan, Laetitia Serrano, Christelle Butel, Rogatien Atoun, Frederic Youa, Komlan Ali-Edje, Messanh Douffan, Phyllis Ehlan, Kokou Tegueni, Romaric Amenti, Abla Ahouefa Konou, Mounerou Salou, Didier K. Ekouevi, Martine Peeters, Anoumou C. Dagnra

**Affiliations:** ^1^National Reference Laboratory for HIV, Viral Hepatitis and Sexually Transmitted Infections (LNR-VIH-HV IST), Lomé, Togo; ^2^National AIDS, Viral Hepatitis and Sexually Transmitted Infections Control Program (PNLS-HV-IST), Lomé, Togo; ^3^African Center of Research in Epidemiology and Public Health (CARESP), Lomé, Togo; ^4^Training and Research Center in Public Health (CFRSP), University of Lomé, Lomé, Togo; ^5^TransVIHMI, Development Research Institute (IRD) / INSERM / University of Montpellier, Montpellier, France; ^6^Molecular Biology and Immunology of Laboratory (BIOLIM-FSS/UL), University of Lomé, Lomé, Togo; ^7^Global Health in the Global South (GHiGS) Team, Bordeaux Population Health Research Center U1219, University of Bordeaux, Inserm, Development Research Institute, Bordeaux, France

**Keywords:** primary resistance, HIV-1, naïve persons, dolutegravir, Togo

## Abstract

Monitoring pre-treatment drug resistance is a priority to assess the emergence of HIV resistance to antiretrovirals, especially in countries where the transition from dolutegravir-based regimens is already effective on a large scale. The aim of this study was to estimate the prevalence of resistance mutations to different classes of antiretrovirals (ARVs) in HIV-1-naïve persons in Togo in 2023. A cross-sectional study was conducted in 30 HIV care centers for people living with HIV in Togo between March and August 2023. Persons newly diagnosed with HIV-1, aged 18 and over and naïve to any ART with a viral load ≥ 6500.00 copies/ml were included for genotypic resistance testing for protease (PR), reverse transcriptase (RT) and integrase (IN) genes using Next-generation sequencing. Mutations were interpreted using the updated Stanford HIVDB and ANRS algorithms after genotypic testing for protease, reverse transcriptase and integrase resistance. Among 376 enrolled *people living with HIV* (PLHIV), resistance analyses were carried out on 321 samples. The median age was 39 years, Interquartile range (IQR) [30-47] and 64.9% were women. No resistance mutations to dolutegravir (DTG) were detected. However, accessory mutations (genetic changes that do not confer significant resistance by themselves) as E157Q/E157EQ (43/57) and T97A/T97TA (14/57) associated with resistance against first-generation integrase inhibitors (INIs) as raltegravir, elvitegravir were found in 17.2% of samples. No major mutations (genetic changes that, on their own, confer high-level resistance of the virus to one or more antiretroviral drugs) were observed in the protease gene. The mutations as T215TS and K70KE (*detected as a mixture*) associated with possible resistance were detected in 0.6% (2/316) for NRTIs and 6.3% (20/316) for NNRTIs. The most frequent NNRTI mutations were K103N (4.4%) and G190A (1.3%). The dominant subtypes were CRF02_AG (64.8%) and CRF06_cpx (12.8%). These results support the continued use of dolutegravir-based regimens in Togo and call for continued monitoring of primary resistance to maintain the effectiveness of therapeutic strategies in Togo.

## 1 Introduction

The introduction of antiretroviral therapy (ART) for HIV has led to a significant reduction in mortality and morbidity among people living with HIV worldwide, particularly in low- and middle-income countries (LMICs) (1, 2). Thanks to therapeutic advances and the implementation of the “test and treat” strategy, treatment of HIV infection has become more accessible and intensified (3, 4). However, this rapid expansion has led to the emergence of antiretroviral resistance profiles, already induced by non-adherence and treatment interruption (5).

Primary HIV antiretroviral resistance is a major obstacle to antiretroviral efficacy, particularly in resource-limited setting where genotypic resistance testing remains poorly accessible (6, 7). Several studies in sub-Saharan Africa have reported variable rates of transmitted resistance, ranging from 2% to over 10% (8, 9). In West Africa, rates of 8.3 and 10.8% have been reported in Niger (10), Togo (11), 4.2% in Morocco (12) and 8.2% in Cameroon (13). Against this backdrop, since 2018, the WHO has recommended the use of dolutegravir as a first- and second-line HIV treatment for all population groups (14–16).

Due to its strong genetic barrier and its effectiveness against viruses, dolutegravir has become as a central pillar of treatment regimens. However, recent studies have shown a non-negligible prevalence of integrase inhibitor (INI) resistance mutations, with dolutegravir-specific resistance being rare. This is one of the examples of study published in Lancet (2023) which revealed INI resistance mutations in 14% and dolutegravir-specific resistance in 6% of persons on dolutegravir-based antiretroviral therapy (15). The second one carried out in Nigeria (2023) and in Sub-Saharan Africa (2024) identified mutations affecting dolutegravir sensitivity, including T66A, G118R, E138K, and R263K (17, 18). The third one called a MeditRes HIV study carried out in 2021 in Mediterranean Europe in newly diagnosed, treatment-naïve persons showed an overall prevalence of INI resistance mutations of 0.23% and a prevalence of resistance to dolutegravir and bictegravir of 0.18%, confirming the rarity of transmission of resistance to second-generation INIs (19). These mutations represent a potential threat, particularly in low-resource countries where access to regular resistance testing remains limited (20–24).

According to the National AIDS, Viral Hepatitis and sexually transmitted infections Control Program (PNLS-HV-IST)_2021 annual report, the integration of dolutegravir as a first, second- and third-line treatment for people living with HIV/Aids (PLHIV) has been effective since 2020 in Togo and by 2022, more than 95% of persons were on a dolutegravir-based regimen (25, 26). Although this massive adoption reflects a significant advance, available data on primary resistance in treatment-naïve persons remain very limited. The aim of this study is to estimate the prevalence of primary HIV-1 resistance to the different classes of antiretroviral drugs and to characterize circulating resistance mutations in HIV-1 initiating a first ART in Togo in 2023.

## 2 Materials and methods

### 2.1 Type and study period

**Figure 1:**
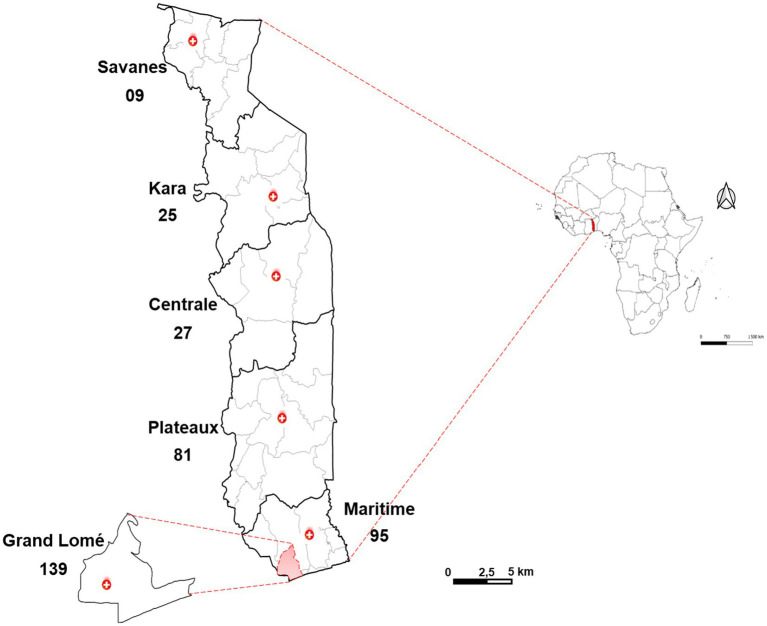
Number of samples collected by health region of Togo (*N* = 376).

A cross-sectional study was carried out between March and August 2023 in 30 screening and care centers for the treatment of AIDS, viral hepatitis and sexually transmitted infections spread across six health regions in Togo (Figure 1).

### 2.2 Study population

The study population is consisted of HIV-1-positive, antiretroviral-naive adults aged 18 and over and all of whom signed a free and informed consent form.

### 2.3 Sample size calculation

- The sample size was estimated on the basis of standardized WHO calculations (23). The following assumptions were made the expected prevalence of primary resistance was set at 10%; the expected proportion of individuals initiating ART with non-nucleoside reverse transcriptase inhibitor (NNRTI)-based regimens was set at 100%;
- the genotyping failure rate has been set at 20%;
- the expected proportion of ART initiators with no prior exposure to antiretrovirals was set at 75%;
- the accuracy of the estimate was set at 5%;
- the minimum number of subjects to be included was 320 HIV-infected persons.

### 2.4 Data collection

Information on socio-demographic characteristics such as age, gender, marital status, date of diagnosis and ART initiation, HIV-1 viral load and planned antiretroviral combination for the person were collected using a questionnaire. ART history was also collected from persons’ medical records.

### 2.5 Laboratory tests

The 2-ml plasma aliquots were stored at −20 °C at the collection sites and then at −80 °C at the national reference laboratory for HIV, viral hepatitis and sexually transmitted infections (LNR-VIH-HV-IST) until used for laboratory testing. Two rapid tests, SD Bioline™ HIV/SYPHILIS Duo (Abbott, Santa Clara, CA, United States) and First Response HIV 1–2. O Card Test version 2.0 (A1-302, GIDC, Sarigam 396,155. Dist. Valsad, Gujarat, India) were used to confirm HIV serology at the sampling sites. A participant was considered HIV-infected when both tests used were positive, in accordance with the national algorithm for HIV testing in Togo (27). Viral RNA was extracted using the GXT NA VER 1.0 extraction kit (BRUKER, Hain Lifescience GmbH) by mixing 250 μL plasma and 10 μL proteinase K. After incubation at room temperature for 10 min, the mixture is transferred to NorDIAG® equipment for extraction. The extraction protocol of 250 μL of plasma to 50 μL of eluate, comprising the steps of lysis, coupling of magnetic beads and RNA, washing and elution, was chosen (28). At the end of extraction, 10 μL of RNA were used to measure HIV-1 viral load using the GENERIC VIH-1 kit (Biocentric, Bandol, France) at the LNR-VIH/IST (29). Under these conditions, the quantification threshold was 390 RNA copies/ml plasma. Samples with viral load value greater than or equal to 6500.00 copies/mL (*N* = 336) were sent to the TransVIHMI—UMI233 laboratory (IRD, Montpellier, France) for resistance genotyping testing. The threshold of 6500.00 copies/mL was selected because it falls within the range of 5000.00 to 10000.00 copies/mL, from which NGS sequencing techniques show significantly higher sensitivity (30). HIV-1 nucleic acids were extracted from 250 μL of eluted in 60 μL of buffer using the magnetic silica Boom method on the Nuclisens EasyMAG automatic extractor (BioMerieux). Nested RT-PCRs using HotStarTaq DNA Polymerase Qiagen GmbH, Germany were performed to separately amplify fragments of the pol gene, coding, respectively, for protease (PR, covering amino acids from position 1 to position 99), part of reverse transcriptase (RT, covering amino acids from position 1 to 257) and part of integrase (IN, covering amino acids from position 37 to position 288) according to the ANRS protocol.^1^ The genes encoding protease, part of reverse transcriptase and part of integrase were amplified by PCR according to the modified ANRS, 2023 protocol, including primers and PCR program. Briefly,

a first-round reverse transcriptase PCR was performed using Invitrogen’s SuperScript IV enzyme (Invitrogen, Carlsbad, CA), PR and RT were amplified using the same PCR program. IN amplification was performed using a different program. Five microliters of the amplicons from the first PCR run were used as template for nested PCR using HotStarTaq DNA Polymerase (Qiagen GmbH, Germany), including the primers and PCR program applied.

Amplification products were visualized by 1% agarose gel electrophoresis with ethidium bromide (BET). The three amplicons (PR, RT, IN) confirmed positive by electrophoresis were grouped inequal quantity for each sample, in a final volume of 60 μL then purified using a 0.6X ratio of Agencourt AMpure XP beads (BECKMAN COULTER, Life Sciences). Approximately 500 ng of product was used for library preparation using the Illumina DNA Prep kit according to the manufacturer’s instructions with average size measured using the Agilent High Sensitivity DNA kit on a Bioanalyzer 2,100 (Agilent Technologies, Santa Clara, CA). Once prepared and verified, the products were sequenced using next generation sequencing (NGS) technology on Illumina (Miseq) platform using the Miseq R v2 500 Cycle Reagent Kit. FASTQ files extracted from the Miseq platform were analyzed using the HIVdb-NGS pipeline,^2^ applying a mutation analysis threshold of 20% to generate consensus sequences in FASTA format. The quality of the sequences was assessed with the Stanford tool. Sequences with a median read depth less than 10,000X were excluded from analysis to ensure sufficient depth to confirm the reliability of the existence of the observed minor mutation (31).

We assessed HIVDR mutations patterns and genotypes’ sensitivity to protease inhibitor (PIs), nucleoside and non-nucleoside reverse transcriptase inhibitors (NRTIs and NNRTIs) and integrase inhibitors (INI) using the Stanford HIVDB algorithm, version HIVDB 9.5.1 (last updated 05/11/2023, available at https://hivdb.stanford.edu/hivdb/by-mutations/).

For analysis of results and study conclusions, mutations were interpreted using the National Agency for Research on AIDS and Viral Hepatitis (ANRS) algorithm (version 35, [April 8,2024]) and classified into three categories: susceptible, confirmed resistance (presence of at least one major mutation) and possible resistance (accessory, polymorphic, or mixture mutations in the absence of major mutations). In cases of coexistence, the classification followed ANRS rules, with priority given to “confirmed resistance” (32). Prevalence was calculated as the proportion of sequences in each category relative to the total number of sequences analyzed (24). Identification of subtypes and circulating recombinant forms (CRF) was carried out using the Stanford site program.

### 2.6 Statistical analysis

Data were entered into Excel and analyzed using R software version 4.2.3 (R Foundation for Statistical Computing, Vienna, Austria). Qualitative variables were summarized as absolute and relative frequencies, along with their 95% confidence intervals (95% CIs) where appropriate. Quantitative variables were presented as medians with their interquartile ranges (IQRs). No comparative or inferential analyses were performed in this study according to the objectives.

### 2.7 Ethical considerations

This study was conducted in accordance with the ethical principles of the Declaration of Helsinki. The study protocol was approved by the National Bioethics Committee for Health Research (CBRS; Number 013/2023/CBRS; Supplementary File 1). Participation in the study was entirely voluntary. Written informed consent was obtained from all enrolled participants prior to data collection. All data were collected and analyzed anonymously to ensure confidentiality and privacy.

## 3 Results

### 3.1 Diagram of inclusions

**Figure 2:**
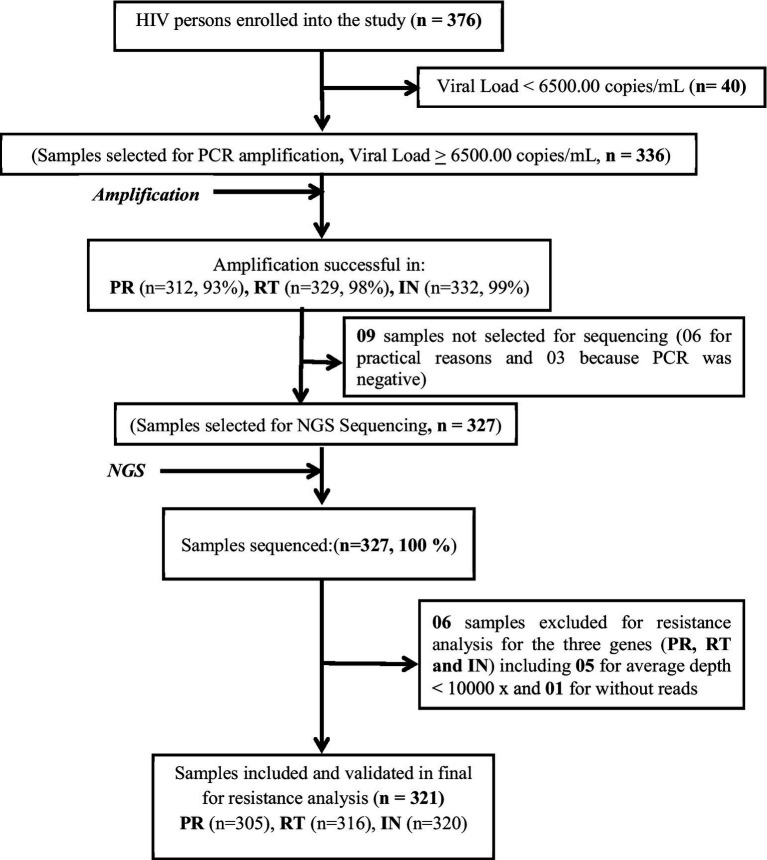
Flow chart for selecting HIV persons for genotyping.

Among the 376 persons enrolled into the study, 40 (10.6%) were excluded for a viral load below 6500.00 copies/mL. At the end, of the 336 selected for PCR amplification of the protease, reverse transcriptase and integrase genes, nine samples were not selected for sequencing (six due to the limited number of places per run on Miseq and three due to failure of the three genes following amplification), i.e. 327 sequenced by NGS. At the end of NGS sequencing and after analysis of the quality of the runs, 321 samples with at least 1 sequenced gene and only 306 samples with the 3 validated PR/RT/IN sequences were selected for resistance analysis (Figure 2).

### 3.2 Socio-demographic and clinical characteristics of participants

The median age was 39 years (IQR = 30–47 years), with 64.9% women. At the time of HIV diagnosis, 62.1% were in stage 1 of the WHO classification (Table 1). In this study, the viral loads of the 336 retained samples ranged from 6500.00 to 1000000.00 copies/mL, with a median of 174000.00 (IQR = [60100.00–621500.00]). The sociodemographic and clinical characteristics are presented in Table 1.

**Table 1:** Socio-demographic characteristics of HIV persons by gender (*N* = 376).

Characteristics	Sex			
	Female	Male	Total	*p*-value
	*N* = 244	*N* = 132	*N* = 376	
Demographics				
Age (years)				
Median (IQR)^1^	38 (29–48)	40 (34–46)	39 (30–47)	0.275^1^
Age ranged (years), n (%)^2^				0.001^**2**^
< 30	62 (25.4)	16 (12.1)	78 (20.7)	
30–49	126 (51.6)	92 (69.7)	218 (58.0)	
≥ 50	56 (23.0)	24 (18.2)	80 (21.3)	
Nationality, n (%)^3^				0.987^3^
Togolese	241 (98.8)	130 (98.5)	371 (98.7)	
Other	3 (1.2)	2 (1.5)	5 (1.3)	
Region of residence, n (%)^3^				0.806^3^
Central	15 (6.1)	12 (9.2)	27 (7.2)	
Grand Lomé	92 (37.7)	47 (35.6)	139 (37.0)	
Kara	18 (7.4)	7 (5.3)	25 (6.6)	
Maritime	63 (25.8)	32 (24.2)	95 (25.3)	
Plateaux	51 (20.9)	30 (22.7)	81 (21.5)	
Savanes	5 (2.1)	4 (3.0)	9 (2.4)	
Socioeconomic				
Study Level, n (%)^2^				<0.001*^2^*
Out of school	69 (28.3)	13 (9.8)	82 (21.8)	
Primary	90 (36.9)	53 (40.2)	143 (38.0)	
Secondary	74 (30.3)	43 (32.6)	117 (31.2)	
University-level	11 (4.5)	23 (17.4)	34 (9.0)	
Type of housing, n (%)^2^				0.131^2^
Collective	172 (70.5)	83 (62.9)	255 (67.8)	
Single	72 (29.5)	49 (37.1)	121 (32.2)	
Place of residence, n (%)^2^				0.611^2^
Rural	119 (48.8)	68 (51.5)	187 (49.7)	
Urban	125 (51.2)	64 (48.5)	189 (50.3)	
Income-generating activity, n (%)^2^				0.024^**2**^
No	68 (27.9)	23 (17.4)	91 (24.2)	
Yes	68 (27.9)	109 (82.6)	285 (75.8)	
Clinical				
Marital status, n (%)^2^				<0.001*^2^*
Single	41 (16.8)	31 (23.5)	72 (19.1)	
Divorced or separated	44 (18.0)	11 (8.3)	55 (14.6)	
Married or cohabiting	120 (49.2)	87 (65.9)	207 (55.1)	
Widowed	39 (16.0)	3 (2.3)	42 (11.2)	
WHO clinical stage, n (%)^3^				0.195^3^
Stage I	155 (63.5)	78 (59.1)	233 (62.1)	
Stage II	73 (29.9)	38 (28.8)	111 (29.5)	
Stage III	13 (5.3)	11 (8.3)	24 (6.4)	
Stage IV	3 (1.3)	1 (0.8)	4 (1.0)	

1 Wilcoxon-Mann–Whitney test.

2 Chi-squared independence test.

3 Fisher exact test.

### 3.3 Prevalence of antiretroviral resistance mutations

#### 3.3.1 Protease inhibitor (PI) resistance

No mutations patterns associated to resistance or possible resistance to protease inhibitors were observed in the 305 samples analyzed were reported according to the ANRS Version 35 algorithm.

#### 3.3.2 Resistance to nucleoside reverse transcriptase inhibitors

For NRTIs, 2 persons out of 316 (0.6%) had two mutations (T215TS and K70KE) conferring possible resistance to tenofovir (TDF) and tenofovir alafenamide (TAF).

#### 3.3.3 Resistance to non-nucleoside reverse transcriptase inhibitors

**Figure 3:**
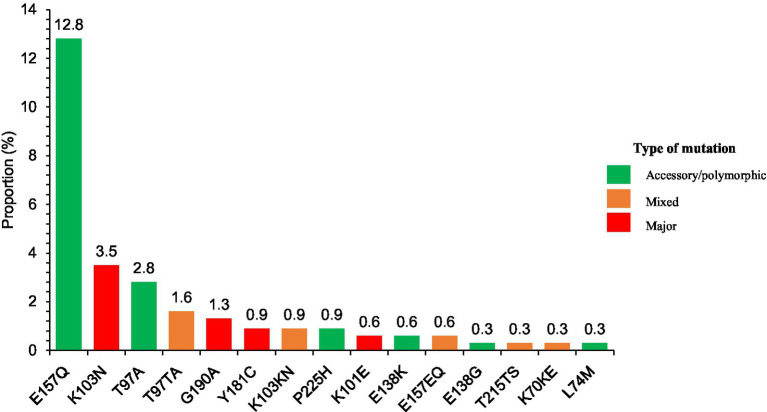
Relative frequency of mutations observed in participants by ART drug classes.

For NNRTIs, 20 persons out of 316 (6.3%) of the sequences studied, had mutations conferring resistance to EFV/NVP (18/316) and NVP (02/316; Supplementary Table 2). The most common resistance mutations were K103N/K103KN in 4.4% (14 strains) and G190A in 1.3% (4 strains) of the RT genes analyzed (Figure 3).

#### 3.3.4 Resistance to integrase inhibitors

The prevalence of integrase inhibitor resistance mutations was 17.8% (57/320). E157Q/E157EQ (43/57) and T97A/T97TA (14/57) mutations induced resistance to Elvitegravir/ Raltegravir (EVG, RAL) or Elvitegravir alone (EVG) respectively, but none were resistant to dolutegravir (DTG). A detailed visualization of the frequency of each resistance-associated mutation detected across the four drug classes is presented in Figure 3.

#### 3.3.5 Prevalence of resistance and possible resistance by therapeutic class

**Figure 4:**
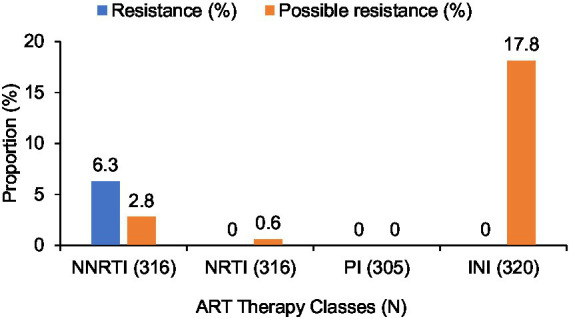
Prevalence of resistance and possible resistance across the four major ART drug classes.

In the overall cohort, the distribution of resistance profiles differed across the four main ARV drug classes (Figure 3). NNRTIs showed the highest frequency of confirmed resistance, with 6.3% of individuals carrying at least one major mutation. No confirmed or possible resistance was detected for protease inhibitors. For integrase inhibitors, no confirmed resistance was identified, but 17.8% of participants presented mutation patterns classified as possible resistance. A complete overview of resistance and possible resistance per drug class is shown in Figure 4.

#### 3.3.6 Genetic diversity of HIV-1

**Figure 5:**
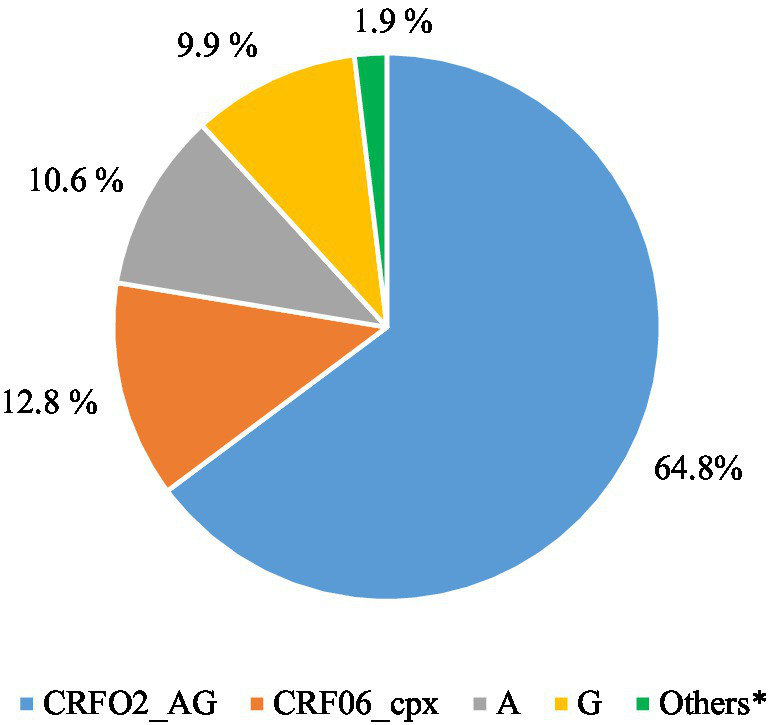
Distribution of HIV-1 subtypes in naive persons with VL ≥ 6500.00 copies/mL based on three gene sequencing (*N* = 321).

Of the 321 samples sequenced and validated, the most common HIV-1 subtypes were CRF02_AG (64.8%) and CRF06_cpx (12.8%). The other subtypes were A (10.6%); G (9.9%); CRF09_cpx (1.6%) and CRF45_cpx (0.3%; Figure 5). All details of the mutations are listed in Supplementary Table 2.

## 4 Discussion

### 4.1 Summary of key results

This study addresses the lack of information on the prevalence of primary HIV-1 resistance mutations among persons initiating antiretroviral therapy (ART) in Togo in the context of widespread use of dolutegravir (DTG). The main finding is the absence of primary resistance to protease inhibitors (PIs) and dolutegravir.

### 4.2 Comparison with previous studies

Our results are consistent with resistance data from other low-resource countries. The absence of PI resistance is comparable to that observed in Vietnam (0%) (33), China (0.2%) (34), Zimbabwe (1%) (35), and Madagascar (0%) (36) likely due to the limited use of PIs as first-line therapy and their high genetic barrier. The observed NRTI resistance rate (0.6%) is lower than those observed in China (3.5%) (34), Vietnam (1.2%) (33), and Zimbabwe (4%) (35). The two mutations detected (T215TS and K70KE) have already been documented in Togo by Dagnra et al. (11), which reinforces the need for continued surveillance given the widespread use of tenofovir in treatment regimens.

In contrast, the prevalence of NNRTI resistance of 6.3% found in our study corresponds to a moderate level according to the WHO classification. This level is above the 5% threshold that justifies increased vigilance in the choice of treatment regimens, but remains below the 15% threshold considered high and requiring a systematic change of treatment lines at the programmatic level (34). These data are consistent with results reported in other countries like in Vietnam (6.3%) (33), in China (3.4%) (34) and in Zimbabwe (15%) (35). The predominance of the K103N mutation, also reported in Madagascar (3.6%) (24), may reflect historical exposure to nevirapine during Prevention of Mother-to-child Transmission of HIV/AIDS (PMTCT) programs, particularly in the predominantly female population of our cohort (33, 34).

It is important to note that no dolutegravir resistance mutations were detected in our study, confirming findings from Zimbabwe and Madagascar (24, 32, 36). However, the detection of the T97A and E157Q mutations, associated with possible resistance against first-generation NNRTIs (e.g., raltegravir, elvitegravir), in treatment-naïve individuals, signals the possibility of transmitted resistance and underscores the need for integrase resistance surveillance in West Africa. Our results are in contrast to those from a pediatric cohort in Togo, reporting high levels of NRTI and NNRTI resistance in children and adolescents receiving DTG-based regimens who did not achieve virological suppression (37).

This discrepancy could be explained in particular by population differences: our study targeted ART-naïve adults, while the cited cohort included HIV-positive adolescents from Prevention of Mother-to-child Transmission of HIV/AIDS (PMTCT) programs who had already received treatment and were experiencing virological failure, a factor that can favor the selection of resistance, particularly in cases of suboptimal adherence or drug exposure.

The high proportion of the CRF02_AG and CRF06_cpx subtypes is consistent with previous findings from Togo (11, 34) and Mali (37, 38). The recently identified CRF45_cpx subtype expands the known molecular epidemiology of HIV in the region and could have implications for diagnostic test performance, vaccine development, and the evolution of drug resistance.

### 4.3 Clinical and public health relevance

These results have several implications. The absence of resistance to dolutegravir (DTG) is reassuring and justifies its continued use as first-line treatment.

However, moderate resistance to NNRTIs and the presence of INSTI mutations warrant systematic resistance monitoring, particularly before initiating antiretroviral therapy, when resources permit.

The integration of basic resistance testing, particularly in high-risk or previously treated populations, could help adapt treatment regimens and reduce the risk of treatment failure (38).

Monitoring genetic diversity is essential to monitor the evolution of circulating strains, anticipate diagnostic challenges, and inform vaccine design (39).

### 4.4 Limitations

In terms of limitations of our study, although we used a high viral load threshold (≥ 6500.00 copies/ml), we were unable to measure antiretroviral drug levels to exclude prior ART exposure, which could overestimate the prevalence of resistance. Second, the lack of phylogenetic analysis limits our understanding of potential transmission chains or resistance clusters. Finally, the cross-sectional nature of the study does not allow conclusions to be drawn on clinical or virological outcomes on the efficacy of dolutegravir-based regimens.

## 5 Conclusion

This national study provides reassuring data supporting the continuation of dolutegravir (DTG)-based regimens as first-line therapy in Togo and justifies its large-scale deployment, particularly in resource-limited settings. However, the considerable prevalence of resistance mutations to first-generation integrase inhibitors, despite the absence of prior ART exposure, raises concerns about pre-existing resistance histories that could impact future integrase-based regimens.

Our results highlight the urgent need to strengthen HIV drug resistance surveillance in Togo, not only among newly diagnosed persons but also among those who have failed treatment. Implementing routine resistance testing and expanding molecular surveillance would enable early detection of emerging resistance patterns, guide individual treatment decisions, and inform national antiretroviral policy. Further studies, integrating ARV dosing, detection of minority variants and phylogenetic analysis, are needed to better understand the transmission dynamics and evolution of drug resistance in West Africa.

## Data Availability

The original contributions presented in the study are included in the article/Supplementary material, further inquiries can be directed to the corresponding author.

## Footnotes

^1^http://www. hivfrenchresistance.org/

^2^ https://hivdb.stanford.edu/hivdb/by-reads/
