# Supplementary material for: Prevalence of primary HIV-1 drug resistance among antiretroviral-naïve individuals in Togo in 2023: a national study
Source: Front Public Health. 2025 Nov 5;13:1605763. doi: 10.3389/fpubh.2025.1605763 (PMC12626816; doi:10.3389/fpubh.2025.1605763)

| **MINISTRY OF HEALTH** | **TOGOLESE REPUBLIC** |
| --- | --- |
| **PUBLIC HYGIENE AND ACCESS** | ***Work—Freedom—Country*** |
| **UNIVERSAL TO CARE** |  |

--------------------- --------------


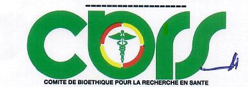


**PROTOCOL EVALUATION OF**

**RESEARCH**

**PROJECT TITLE: “Study of HIV resistance to ARVs in PLHIV initiating first antiretroviral treatment in Togo in 2023 »**

**Protocol date**: Version not indicated

**Promoter**: PNLS/HV/IST

**Consultant:** CARESP Cabinet

**Technical team**:

- **Pr EKOUEVI Koumavi Didier**, principal investigator **Dr PATASSI Akouda**, associate investigator
- **Dr. NYASENU Xavier**, biology investigator
- **Dr. TOGAN Roméo**, Project manager
- **Dr YOUA Yacoubou Iman**, clinical study monitor **Dr ATOUN Comlan Rogatien**, clinical study monitor
- **Dr BESSIKE KOISSI Abiré Adèle**, clinical study monitor **Dr ADEDJOUMA Taissiri**, clinical study monitor
- **Dr. SATO Aléki**, clinical study monitor  **Mr. TCHANKONI Martin**, biostatistician
- **Mr. HALIBA Jérémie**, biostatistician assistant.

**NOTICE NO.
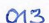
_/2023/CBRS of March 23, 2023**

The Bioethics Committee for Health Research (CBRS) met on March 23, 2023 to evaluate the research protocol relating to the following study: “**Study of HIV resistance to ARVs in PLHIV initiating first antiretroviral treatment in Togo in 2023**”.

- Following the pooling of study reports presented by resource persons, the CBRS ruled on:

1. **Presentation of the file**

- Study protocol documents: The documents are well presented.
- Data collection tools are available and well developed.

**2. The scientific validity of the file**


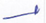


| **MINISTRY OF HEALTH** | **TOGOLESE REPUBLIC** |
| --- | --- |
| **PUBLIC HYGIENE AND ACCESS** | ***Work—Freedom—Country*** |
| **UNIVERSAL TO CARE** |  |

--------------------- --------------


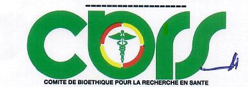


**Scientific design**: The study is scientifically well designed.

WHO recommends that countries with an ARV treatment program implement a strategy to prevent HIV resistance to these drugs. One of the activities of this strategy is the regular monitoring of the level of HIV resistance to ARVs used in the country by PLHIV. Within this framework, Togo has been conducting studies on HIV resistance to ARVs for several years in order to optimize the quality of treatment protocols. This study is part of this series of regular monitoring conducted by the national AIDS control program.

**Hypothesis**: Problematic AI is well defined: There are strains of Virus resistant to antiretrovirals in PLHIV who are not yet taking these drugs.

**Methodology**: The methodology is well described and is suitable for this type of study. It is a single-pass, quantitative cross-sectional survey. It is suitable for this type of study. Determination of study areas and sites: All regions are covered.

Target populations of the study identified: PLHIV initiating the first treatment.

- CD4 and Viral Load Testing in Lomé
- ARV drug dosage and strain genotyping in Montpellier, France.

**Feasibility:** This protocol is feasible.

**Objectives**: The objectives are clear and precise.

**Interest of the study**: The interest of this work for public health is undeniable.

**References scientists**: Scientific references are provided.

**3. Ethical acceptability**

The participant information leaflet and consent form are available.


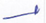


| **MINISTRY OF HEALTH** | **TOGOLESE REPUBLIC** |
| --- | --- |
| **PUBLIC HYGIENE AND ACCESS** | ***Work—Freedom—Country*** |
| **UNIVERSAL TO CARE** |  |

--------------------- --------------


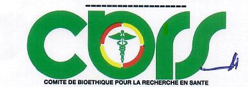


**4. The conclusion**

The CBRS, unanimously by its members present, issued a **FAVORABLE OPINION** for the execution in Togo of the following study protocol: “**Study of HIV resistance to ARVs in PLHIV initiating a first antiretroviral treatment in Togo in 2023**”.

NB: At the end of the study, the promoter must submit 5 copies of the final report to the CBRS secretariat.

Made in Lome, March 23th, 2023

**The Rapporteur:**


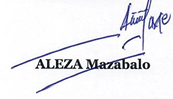

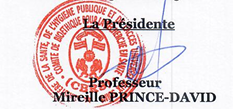

Supplement: Supplementary file 1 [file Supplementary_file_1.docx]
