# Supplementary material for: Prevalence of primary HIV-1 drug resistance among antiretroviral-naïve individuals in Togo in 2023: a national study
Source: Front Public Health. 2025 Nov 5;13:1605763. doi: 10.3389/fpubh.2025.1605763 (PMC12626816; doi:10.3389/fpubh.2025.1605763)
Supplement: Supplementary file 2 [file Supplementary_file_2.docx]

***Supplementary file 2 /Table 2 : Major and accessory mutations observed and prediction of resistance to antiretrovirals ARVs according to the ANRS algorithm (Version 35 of April 8, 2024)***

| **Person code** | **Mutations NRT** | | **Mutations NNRT** | | **Mutations IN** | | **Subtypes CRF** | **ARV resistance** |
| --- | --- | --- | --- | --- | --- | --- | --- | --- |
| **Type of mutation** | *Major mutation* | *Accessory/Polymorphic /mixed mutation* | *Major mutation* | *Accessory/Polymorphic /mixed mutation* | *Major mutation* | *Accessory/Polymorphic /mixed mutation* |  |  |
| RP001 | Not detected | Not detected | Not detected | Not detected | Not detected | T97A | CRF02_AG | EVG |
| RP002 | Not detected | Not detected | Not detected | Not detected | Not detected | E157Q | CRF02_AG | EVG, RAL |
| RP003 | Not detected | Not detected | Not detected | Not detected | Not detected | T97A | CRF02_AG | EVG |
| RP020 | Not detected | Not detected | Not detected | Not detected | Not detected | E157Q | A | EVG, RAL |
| RP026 | Not detected | Not detected | K103N, G190A, Y181C | Not detected | Not detected | Not detected | G | EFV, NVP, ETR, RPV, DOR |
| RP027 | Not detected | Not detected | K101E, G190A | E138K | Not detected | Not detected | CRF02_AG | EFV, NVP, ETR, RPV |
| RP036 | Not detected | Not detected | Not detected | Not detected | Not detected | E157Q | CRF06_cpx | EVG, RAL |
| RP037 | Not detected | Not detected | Not detected | Not detected | Not detected | T97A | CRF06_cpx | EVG |
| RP049 | Not detected | Not detected | K103N | Not detected | Not detected | E157Q | CRF02_AG | EFV, NVP, EVG, RAL |
| RP057 | Not detected | Not detected | Not detected | Not detected | Not detected | E157Q | CRF02_AG | EVG, RAL |
| RP059 | Not detected | Not detected | Not detected | Not detected | Not detected | T97TA | CRF02_AG | EVG |
| RP067 | Not detected | Not detected | Not detected | Not detected | Not detected | E157Q | CRF02_AG | EVG, RAL |
| RP070 | Not detected | Not detected | K103N | Not detected | Not detected | Not detected | CRF02_AG | EFV, NVP |
| RP071 | Not detected | Not detected | Not detected | K103KN | Not detected | Not detected | CRF02_AG | EFV, NVP |
| RP076 | Not detected | Not detected | Not detected | Not detected | Not detected | T97A, E157Q | CRF02_AG | EVG, RAL |
| RP079 | Not detected | Not detected | Not detected | Not detected | Not detected | T97TA | CRF06_cpx | EVG |
| RP085 | Not detected | Not detected | Not detected | Not detected | Not detected | E157Q | CRF02_AG | EVG, RAL |
| RP091 | Not detected | Not detected | Not detected | Not detected | Not detected | E157Q | CRF02_AG | EVG, RAL |
| RP101 | Not detected | Not detected | K103N | E138G | Not detected | Not detected | CRF02_AG | EFV, NVP, RPV |
| RP108 | Not detected | Not detected | Not detected | Not detected | Not detected | E157Q | CRF02_AG | EVG, RAL |
| RP114 | Not detected | Not detected | G190A | Not detected | Not detected | Not detected | CRF02_AG | NVP |
| RP117 | Not detected | Not detected | Not detected | Not detected | Not detected | E157Q | CRF02_AG | EVG, RAL |
| RP123 | Not detected | Not detected | K103N | Not detected | Not detected | Not detected | CRF02_AG | EFV, NVP |
| RP126 | Not detected | Not detected | Not detected | Not detected | Not detected | T97TA | CRF02_AG | EVG |
| RP128 | Not detected | Not detected | Not detected | Not detected | Not detected | T97A | CRF02_AG | EVG |
| RP142 | Not detected | Not detected | Not detected | Not detected | Not detected | E157Q | CRF02_AG | EVG, RAL |
| RP143 | Not detected | Not detected | Not detected | Not detected | Not detected | E157Q | CRF02_AG | EVG, RAL |
| RP144 | Not detected | Not detected | Not detected | Not detected | Not detected | E157Q | CRF02_AG | EVG, RAL |
| RP166 | Not detected | Not detected | Not detected | Not detected | Not detected | E157Q | CRF02_AG | EVG, RAL |
| RP173 | Not detected | Not detected | K103N | P225H | Not detected | E157Q | CRF02_AG | EFV, NVP, EVG, RAL |
| RP177 | Not detected | Not detected | K103N | Not detected | Not detected | Not detected | CRF02_AG | EFV, NVP |
| RP187 | Not detected | Not detected | Not detected | Not detected | Not detected | E157Q | CRF02_AG | EVG, RAL |
| RP193 | Not detected | Not detected | Not detected | Not detected | Not detected | E157Q | CRF02_AG | EVG, RAL |
| RP198 | Not detected | Not detected | Not detected | Not detected | Not detected | T97TA | CRF02_AG | EVG |
| RP205 | Not detected | Not detected | Not detected | Not detected | Not detected | E157Q | CRF09_cpx | EVG, RAL |
| RP207 | Not detected | Not detected | Y181C | Not detected | Not detected | Not detected | CRF02_AG | EFV, NVP, ETR, RPV |
| RP209 | Not detected | Not detected | Not detected | Not detected | Not detected | E157Q | CRF02_AG | EVG, RAL |
| RP222 | Not detected | Not detected | Not detected | Not detected | Not detected | E157Q | CRF06_cpx | EVG, RAL |
| RP243 | Not detected | Not detected | Not detected | Not detected | Not detected | E157EQ | CRF02_AG | EVG, RAL |
| RP257 | Not detected | Not detected | Not detected | Not detected | Not detected | E157Q | CRF02_AG | EVG, RAL |
| RP259 | Not detected | Not detected | K103N | P225H | Not detected | Not detected | CRF06_cpx | EFV, NVP |
| RP262 | Not detected | Not detected | Not detected | Not detected | Not detected | E157Q | CRF02_AG | EVG, RAL |
| RP263 | Not detected | Not detected | Not detected | Not detected | Not detected | E157Q | CRF02_AG | EVG, RAL |
| RP264 | Not detected | Not detected | Not detected | Not detected | Not detected | E157Q | CRF02_AG | EVG, RAL |
| RP266 | Not detected | Not detected | Not detected | Not detected | Not detected | E157Q | CRF02_AG | EVG, RAL |
| RP277 | Not detected | Not detected | Not detected | Not detected | Not detected | L74M, T97A | CRF02_AG | EVG |
| RP278 | Not detected | Not detected | Not detected | Not detected | Not detected | E157Q | CRF09_cpx | EVG, RAL |
| RP288 | Not detected | Not detected | G190A | Not detected | Not detected | Not detected | G | NVP |
| RP303 | Not detected | Not detected | Not detected | Not detected | Not detected | T97A, E157Q | CRF02_AG | EVG, RAL |
| RP304 | Not detected | Not detected | K103N, Y181C | Not detected | Not detected | Not detected | CRF06_cpx | EFV, NVP, ETR, RPV |
| RP305 | Not detected | T215TS | Not detected | Not detected | Not detected | E157Q | CRF02_AG | TDF, TAF, EVG, RAL |
| RP314 | Not detected | Not detected | Not detected | Not detected | Not detected | E157Q | CRF02_AG | EVG, RAL |
| RP319 | Not detected | Not detected | Not detected | Not detected | Not detected | E157Q | CRF02_AG | EVG, RAL |
| RP324 | Not detected | Not detected | K103N | Not detected | Not detected | Not detected | CRF06_cpx | EFV, NVP |
| RP330 | Not detected | Not detected | Not detected | Not detected | Not detected | E157Q | CRF02_AG | EVG, RAL |
| RP331 | Not detected | Not detected | K103N | Not detected | Not detected | E157Q | G | EFV, NVP |
| RP335 | Not detected | K70KE | Not detected | Not detected | Not detected | Not detected | CRF09_cpx | TDF, TAF |
| RP336 | Not detected | Not detected | Not detected | Not detected | Not detected | E157Q | CRF02_AG | EVG, RAL |
| RP339 | Not detected | Not detected | Not detected | Not detected | Not detected | E157Q | CRF06_cpx | EVG, RAL |
| RP341 | Not detected | Not detected | Not detected | K103KN**,** P225H | Not detected | Not detected | CRF02_AG | EFV, NVP, DOR |
| RP346 | Not detected | Not detected | Not detected | Not detected | Not detected | T97A | A | EVG |
| RP347 | Not detected | Not detected | Not detected | Not detected | Not detected | T97TA | CRF02_AG | EVG |
| RP350 | Not detected | Not detected | Not detected | Not detected | Not detected | E157Q | CRF02_AG | EVG, RAL |
| RP360 | Not detected | Not detected | K103N | Not detected | Not detected | Not detected | CRF02_AG | EFV, NVP |
| RP379 | Not detected | Not detected | Not detected | Not detected | Not detected | E157Q | CRF02_AG | EVG, RAL |
| RP389 | Not detected | Not detected | Not detected | Not detected | Not detected | E157Q | CRF02_AG | EVG, RAL |
| RP408 | Not detected | Not detected | Not detected | Not detected | Not detected | T97A | CRF02_AG | EVG |
| RP425 | Not detected | Not detected | Not detected | Not detected | Not detected | E157Q | CRF02_AG | EVG, RAL |
| RP426 | Not detected | Not detected | Not detected | Not detected | Not detected | E157Q | CRF02_AG | EVG, RAL |
| RP427 | Not detected | Not detected | Not detected | Not detected | Not detected | E157EQ | CRF02_AG | EVG, RAL |
| RP430 | Not detected | Not detected | Not detected | Not detected | Not detected | E157Q | CRF02_AG | EVG, RAL |
| RP433 | Not detected | Not detected | K101E | E138K | Not detected | Not detected | CRF06_cpx | EFV, NVP |

*TDF/TAF: tenofovir alafenamide, EFV: efavirenz, NVP: nevirapine, EVG: elvitegravir, RAL: raltegravir, ETR: etravirine, RPV: rilpivirine, DOR: doravirine.*
